# Vitamin A status is associated with sleep, clock genes, and symptoms in children with autism spectrum disorder

**DOI:** 10.3389/fpsyt.2026.1805599

**Published:** 2026-04-07

**Authors:** Xueli Xiang, Hongyu Chen, Binlin Yuan, Qiuhong Wei, Binyue Hu, Danyang Zhang, Dan Ai, Ting Yang, Jie Chen, Tingyu Li, Yuan Ding

**Affiliations:** Growth, Development and Mental Health Center of Children and Adolescents, Chongqing Key Laboratory of Child Neurodevelopment and Cognitive Disorders, National Clinical Research Center for Children and Adolescents’ Health and Diseases, Ministry of Education Key Laboratory of Child Development and Disorders, Children’s Hospital of Chongqing Medical University, Chongqing, China

**Keywords:** autism spectrum disorder, clock genes, core symptoms, sleep problems, vitamin A

## Abstract

**Background:**

Vitamin A signals through retinoic acid receptors and may influence neurodevelopment and the expression of clock genes. However, the biological pathway linking vitamin A status to sleep disturbance in ASD remains insufficiently defined. This study aimed to examine associations between vitamin A status and sleep problems, core symptoms, and clock genes in children with ASD, and to explore the mechanistic role of RARβ in regulating core clock genes.

**Methods:**

This observational study included 361 children with ASD. Clinical symptoms were assessed using the Children’s Sleep Habits Questionnaire (CSHQ); the Childhood Autism Rating Scale (CARS) and the Social Responsiveness Scale (SRS). Peripheral blood mononuclear cell (PBMC) mRNA levels of RARβ and clock genes (BMAL1 and CLOCK) were quantified by qPCR. RARβ expression was knocked down in mice by stereotaxic injection of adeno-associated virus.

**Results:**

Children with lower vitamin A levels exhibited more severe sleep problems and autistic symptoms. Vitamin A levels showed a weak positive correlation with the expression of RARβ and BMAL1. RARβ knockdown reduced the expression of RARβ and clock genes in mouse brain tissue. Chromatin immunoprecipitation quantitative PCR (ChIP-qPCR) confirmed RARβ occupancy at a predicted CLOCK regulatory region.

**Conclusion:**

This study provided evidence that vitamin A status was linked to sleep problems, symptom severity, and expression of clock genes in the morning in ASD. We also found that RARβ signaling may regulate the expression of clock genes. This finding provides new insights into the mechanisms underlying sleep disturbances in ASD, but further functional studies are needed to confirm these findings.

## 1 Introduction

Autism spectrum disorder (ASD) is a complex neurodevelopmental disorder characterized by impaired social communication, restricted interests, and stereotypic behaviors (1). Beyond these core symptoms, children with ASD frequently experience sleep disturbances and nutritional deficiencies, which further exacerbate societal and familial burdens.

Sleep plays critical roles in brain development, cognition, and emotion (2), governed primarily by two mechanisms: sleep homeostasis and the circadian rhythm (3). CLOCK and BMAL1 represent core clock genes whose encoded proteins form heterodimers. These bind to E-box elements within gene promoter regions to initiate transcription of downstream clock-controlled genes. Previous studies reported a comorbidity rate exceeding 50% for sleep problems in children with ASD. These disturbances not only compromised sleep integrity but also contributed to daytime sleepiness, behavioral fluctuations, and exacerbation of core symptoms. Consequently, effectively managing sleep disturbances in ASD is critical for enhancing their quality of life and mitigating core symptoms.

Vitamin A influences neuronal development through the retinoic acid signaling pathway (4, 5) and has been linked to the pathogenesis of ASD (6, 7). Previous studies indicated that nutrients play significant roles in sleep regulation (8), but the relationship between vitamin A and sleep remains unclear. Only one study compared VA levels between those with and without sleep problems, finding no significant difference (9). Several animal studies have suggested an association between VA and sleep. For example, Vitamin A deficiency (VAD) altered sleep electroencephalography (EEG) in mice, with restoration to normal patterns upon VA supplementation (VAS) (10); VAD disrupted molecular oscillations of circadian rhythm molecules in the rat hippocampus (11, 12).

These findings imply potential VA-sleep interactions. Therefore, this study aimed to: (1) investigate the association between VA, sleep problems and core symptoms in children with ASD; (2) investigate the association between VA and the expression of RARβ and clock genes (CLOCK and BMAL1) measured in the morning; (3) explore whether downregulation of RARβ signaling is associated with altered brain clock gene expression and ASD-relevant social behavior.

## 2 Materials

### 2.1 Study design and ethical procedures

This observational study was conducted in Chongqing, China, from November 2019 to December 2024. The study received ethical approval from the Children’s Hospital of Chongqing Medical University Ethics Committee [approval number: 121-1/2018]. Written informed consent was obtained voluntarily from all participants’ parents.

### 2.2 Study participants

Children with ASD were recruited from developmental-behavioral pediatric outpatient departments and the Specialized Learning Center. Inclusion criteria: 1) age 2–7 years, 2) diagnosis confirmed by an experienced developmental-behavioral pediatrician using Diagnostic and Statistical Manual of Mental Disorders, Fifth Edition (DSM-5) criteria, and 3) signed informed consent from primary caregivers. Exclusion criteria: 1) severe physical, neurological, or psychiatric comorbidities; 2) acute/chronic infection within 3 months; 3) incomplete questionnaire data.

### 2.3 Clinical measures of children with ASD

Standardized questionnaires were used to collect baseline information on children with ASD, including: 1) gender and birth date, 2)individual and familial history, and 3) nutritional supplementation in the last 3 months.

Sleep disturbances were assessed using the Children’s Sleep Habits Questionnaire (CSHQ) (13). It was completed by primary caregivers based on the child’s sleep patterns during a typical week in the last month. The eight dimensions of CSHQ were bedtime resistance, sleep disordered breathing, daytime sleepiness, sleep anxiety, sleep duration, sleep onset delay, night wakings, and parasomnias. Based on a previous study (14), these subscales were categorized as medically based or behaviorally based sleep problems. According to recent studies, a total score≥ 48 suggests a sleep problem (15–17).

Core symptoms of ASD were assessed by the Childhood Autism Rating Scale (CARS) (18) and the Social Responsiveness Scale (SRS) (19). The CARS scale evaluates ASD symptom severity through 15 items, each scored on a 4-point scale (1-4) (20). The SRS scale reflects the social behavior of ASD and contains 65 items, with each item scored between 0 and 3. It was divided into social awareness, social cognition, social communication, social motivation, and autistic mannerisms.

### 2.4 Lab measurements

Children in the study underwent fasting venous blood collection between 8:00 and 10:00 AM. Vitamin A levels were quantified using High Performance Liquid Chromatography-Tandem Mass Spectrometry (HPLC-MS/MS). Vitamin A normal (VAN): ≥ 0.3 mg/L; Marginal Vitamin A deficiency (MVAD): 0.2-0.3 mg/L; Vitamin A deficiency (VAD): < 0.2 mg/L. The blood cells were used to extract total RNA, using the Trizol method. The PrimeScript RT reagent Kit (TaKaRa) was used for reverse transcription. Real-time quantitative polymerase chain reaction was employed to assess the relative mRNA expression of CLOCK, BMAL1, and RARβ. Primer sequences were designed using Primer 6.0 software (Premier Biosoft International) and synthesized by the Beijing Genomics Institute. Primer sequences are listed in **Supplementary Table 1**.

### 2.5 Animals

We obtained C57BL/6 mice from Chongqing Enswell Biologicals and housed them in Specific pathogen free (SPF) grade environment. Adeno-associated virus (AAV) was injected stereotaxically into the lateral ventricle of 3-week-old mice. They were divided into a negative control group (short hairpin Negative Control RNA, sh-NC) and a down-regulated RARβ group (short hairpin RNA targeting RARβ, sh-RARβ). All animal experiments complied with the Guidelines for the Care and Use of Laboratory Animals developed by the National Research Council and approved by the Ethics Committee for Animal Experiments of Chongqing Medical University (CHCMU-IACUC20250217007).

### 2.6 Behavioral tests and laboratory analyses in animals

Behavioral tests were performed when mice were reared until 7 weeks of age, including three-chamber and open-field tests. We utilized the three-chamber test (a commonly used indicator of socialization in mice) to assess perceived social novelty and social interactions in mice (13). The open-field test was used to assess exploratory behavior and repetitive tendencies of mice in a novel environment (14). Recordings were made using the ANY-Maze Animal Behavioral Video Analysis System (ANY-Maze, USA).

Mice were reared until 8 weeks of age for tissue collection (prefrontal cortex, PFC), with all sampling performed between 8:00 and 9:00 AM. Total RNA from the mouse PFC was extracted using the Bioer Simply P Total RNA Extraction Kit according to the manufacturer’s protocol. RT-PCR and qPCR procedures followed the methodology described in the human cohort section, with primer sequences provided in **Supplementary Table 2**. The total protein in PFC was extracted using a radio immunoprecipitation assay (RIPA) lysis buffer (KeyGEN Biotech) containing 0.1% protease inhibitor cocktail (KeyGEN Biotech). The protein concentrations were determined using the BCA protein assay kit (KeyGEN Biotech). Western blotting was performed to detect the protein expression levels of RARβ (HuaBio), CLOCK (HuaBio), and BMAL1 (HuaBio). The JASPAR database was used to predict binding sites of the transcription factor RARβ within the mouse Clock gene promoter region, with the two highest relative score sites selected for subsequent experiments (predicted sites detailed in **Supplementary Table 3**). Chromatin immunoprecipitation (ChIP) assays were conducted using the ChIP kit (Abclonal), with qPCR quantification of ChIP results. Primers targeting the predicted binding sites were designed as specified in **Supplementary Table 4**.

### 2.7 Statistical analysis

Statistical analysis and graphing were performed using R-4.4.1 and GraphPad Prism 8.0. Normality was assessed using the Kolmogorov-Smirnov (KS) and Shapiro-Wilk (SW) tests combined with graphical methods (histograms, PP plots, and QQ plots). Continuous variables were described as mean ± standard deviation (M ± SD) or median (25th to 75th percentile) [M (P25-P75)], depending on whether they conformed to normal distribution. Categorical variables were described as frequency (percentage) [n, (%)]. For between-group comparisons, two independent samples t-test was used for comparison of two groups conforming to normal distribution; Mann-Whitney U test was used for non-normally distributed data; The chi-square test was used for categorical variables. McNemar’s test was used for categorical variables. Linear regression analysis was used for the association with VA, sleep scores and autistic symptoms. Spearman correlation analyse was used to assess the relationship among vitamin A, RARβ, and clock gene expression. All statistical tests were two-sided, with *P* < 0.05 considered statistically significant.

## 3 Results

### 3.1 Study population

The study cohort included 361 children diagnosed with ASD and aged 2–7 years. They were enrolled from 2019 to 2024 in Chongqing (**Table 1**).

**Table 1:** Demographics in children with ASD.

Variables	ASD (n=361)
Age (years), M (P25-P75)	3.88 (3.23, 4.57)
Gender, n (%)	
Male	295 (81.7)
Female	66 (18.3)
Ethnicity, n(%)	
Han	329 (91.1)
Others	32 (8.9)
picky about food, n(%)	
Yes	125 (35.2)
No	230 (64.8)
Residence, n(%)	
City	320 (88.6)
Countryside	41 (11.4)
Mother’s education level, n(%)	
Middle school or below	62 (17.2)
High school	89 (24.7)
College	192 (53.1)
Master degree or above	13 (3.6)
Missing	5 (1.4)
Father’s education level, n(%)	
Middle school or below	64 (17.8)
High school	82 (22.7)
College	193 (53.5)
Master degree or above	17 (4.7)
Missing	5 (1.3)
Annual family income, n(%)	
< 60,000	90 (24.9)
60,000 to 150,000	200 (55.4)
≥ 150,000	63 (17.5)
Missing	8 (2.2)

ASD: Autism spectrum disorder; M (P25-P75): Median (25th percentile to 75th percentile);.

Data was shown as M (P25-P75) or n (%).

### 3.2 Association between vitamin A levels, CSHQ dimension scores and ASD symptoms

After adjusting for age and gender and applying FDR correction, vitamin A levels in children with ASD were negatively correlated with bedtime resistance (β=-4.188, 95%CI: -7.072, -1.305, FDR q=0.041) and social awareness (β=-5.355, 95%CI: -9.248, -1.462, FDR q=0.043). Although other sleep dimensions and ASD symptoms did not reach statistical significance, they all exhibited a trend toward negative correlation (**Table 2**).

**Table 2:** Association between vitamin A levels, CSHQ dimension scores and ASD symptoms.

Variables	β(95%CI)	Raw *P*	FDR q
Sleep scores			
Bedtime resistance	-4.188 (-7.072, -1.305)	**0.005**	**0.041**
Sleep onset delay	-0.578 (-1.531, 0.375)	0.234	0.526
Sleep duration	-0.809 (-2.642, 1.023)	0.386	0.579
Sleep anxiety	-0.806 (-2.996, 1.384)	0.470	0.604
Night waking	-0.138 (-1.274, 0.997)	0.811	0.811
Parasomnia	-0.277 (-2.219, 1.666)	0.780	0.811
Sleep-disordered breathing	-0.409 (-1.303, 0.486)	0.370	0.579
Daytime sleepiness	-2.848 (-6.473, 0.777)	0.123	0.370
Total score	-9.346 (-17.115, -1.577)	0.019	0.083
SRS			
Social awareness	-5.355 (-9.248, -1.462)	**0.007**	**0.043**
Social cognition	-2.936 (-8.613, 2.741)	0.310	0.465
Social communication	-8.527 (-19.825, 2.771)	0.139	0.317
Social motivation	-2.02 (-9.196, 5.156)	0.580	0.580
Autism behavior mannerisms	-3.131 (-11.123, 4.861)	0.442	0.530
SRS total score	-21.968 (-52.555, 8.619)	0.159	0.317
CARS total score	-1.349 (-9.334, 6.636)	0.740	0.740

ASD, autism spectrum disorder; β (95% CI), regression coefficient (95% confidence interval), FDR: False Discovery Rate. Linear regression adjusted for age and sex. An FDR threshold of <0.05 was applied to identify significant associations. Bold values indicate *P* < 0.05.

### 3.3 Association between vitamin A, RARβ, and clock genes

As shown in **Table 3**, VA levels in children with ASD exhibited a weak positive correlation with RARβ(ρ=0.112, P = 0.034) and BMAL1 (ρ=0.165, P = 0.002). RARβ was also weakly correlated with CLOCK (ρ=0.211, P<0.001) and BMAL1 (ρ=0.299, P<0.001). Although these correlations are weak, they suggest a potential link between VA, RARβ, and clock genes. Therefore, we further explored their association in a RARβ knockdown animal model.

**Table 3:** Association between vitamin A, RARβ, and circadian rhythm molecules.

Variables	VA		RARβ	
	*ρ*	*P*	*ρ*	*P*
RARβ	0.112	**0.034**		
CLOCK	0.044	0.408	0.211	**<0.001**
BMAL1	0.165	**0.002**	0.299	**<0.001**

ρ: Spearman correlation coefficient. Bold values indicate *P* < 0.05.

### 3.4 Results of behavioral tests, mRNA and protein expression levels, and CHIP-qPCR in sh-NC and sh-RARβ groups of mice

**Figure 1:**
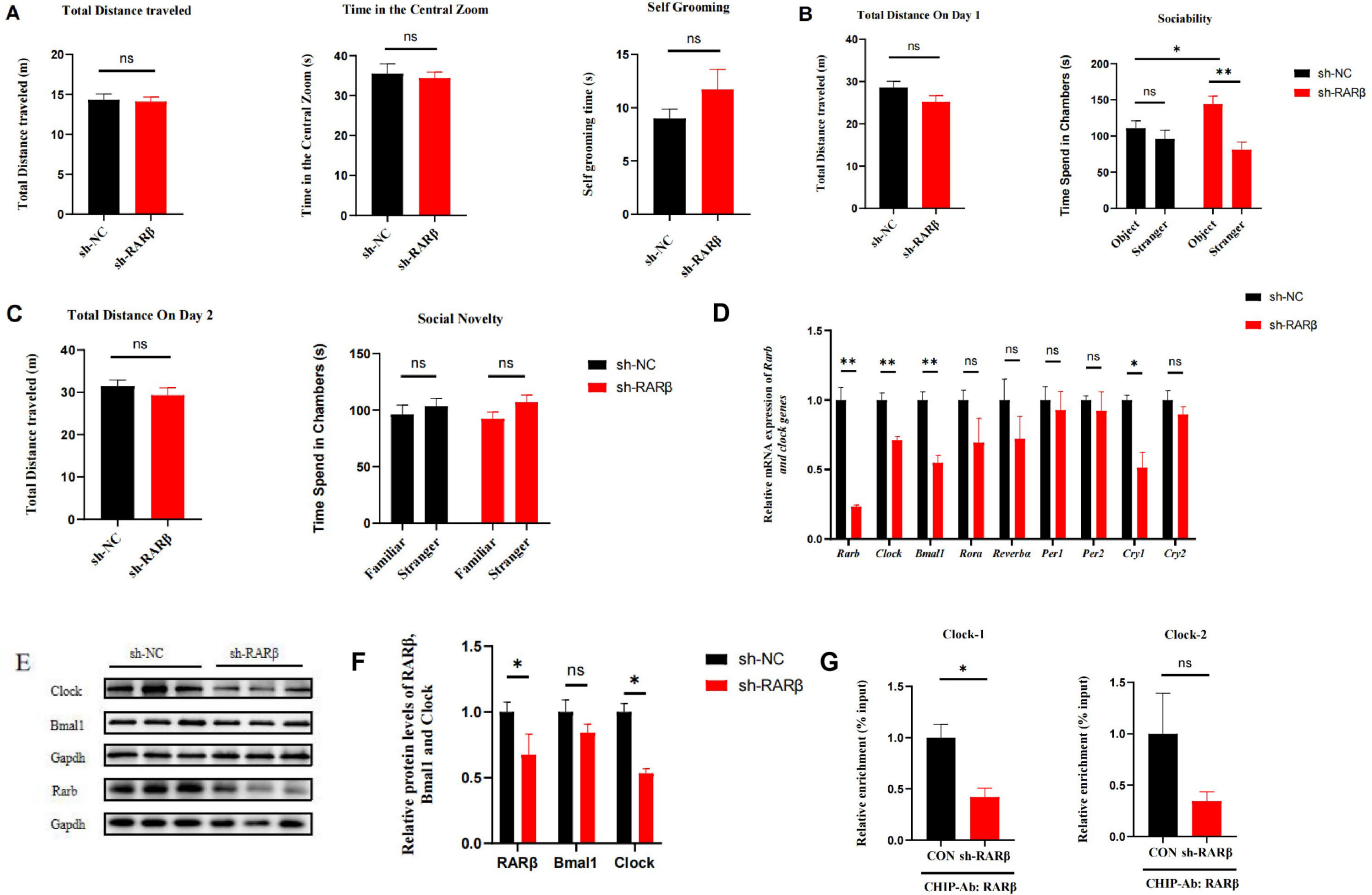
Results of behavioral tests, mRNA and protein expression levels, and CHIP-qPCR in sh-NC and sh-RARβ groups of mice. **(A)** Total distance traveled, duration in the center zone, and self-grooming duration in the open field test; **(B, C)** Duration in the object and stranger mouse zones on the first and second days in the three-chamber test; **(D)** mRNA expression levels of RARβand circadian rhythm molecules in the prefrontal cortex of the two groups of mice; **(E, F)** Protein expression levels of RARβ and circadian rhythm molecules in the prefrontal cortex of the two groups of mice; **(G)** Chromatin immunoprecipitation quantitative PCR (ChIP-qPCR) analysis of RARβ binding to the Clock gene promoter *P<0.05, **P<0.01.

We conducted preliminary experiments to investigate the regulatory mechanism of the retinoic acid receptor RARβ on clock genes. We down-regulated RARβ signaling in the mouse PFC by brain stereotaxic injection of AAV. In the open-field test, no statistically significant differences were observed between the sh-RARβ and sh-NC groups in total distance moved, time spent in the center zone, or self-grooming duration (**Figure 1A**, all P > 0.05). In the three-chamber test, the sh-RARβ group spent significantly more time in the object zone than in the stranger mouse zone on day one. Furthermore, their time in the object zone was significantly longer than that of the control group (**Figures 1B, C**, all P < 0.05), suggesting impaired social interaction in the sh-RARβ group. These results indicate that down-regulating RARβ signaling in the mouse PFC may impair social interaction ability. Additionally, cortical mRNA levels of RARβ, Clock, Bmal1, and Cry2 were significantly decreased in the sh-RARβ group compared to controls (all P < 0.05). Clock protein levels were also significantly reduced (P < 0.05), while Bmal1 protein expression showed a non-significant decreasing trend (P > 0.05) (**Figures 1D–F**).

ChIP-qPCR was performed to assess the binding of RARβ to the predicted binding sites within the Clock gene promoter region in the mouse PFC. The results showed that in the mouse PFC, there was a significant difference in the binding of transcription factor RARβ at predicted site 1 of the Clock promoter region of the target gene, with a significant decrease in the binding of the sh-RARβ group (P < 0.05, **Figure 1G**). Binding enrichment at predicted site 2 also showed a decrease in the sh-RARβ group, although this difference was not statistically significant (**Figure 1G**). These preliminary findings suggest that the transcription factor RARβ may regulate Clock gene expression by specifically binding to Site 1 in its promoter region. However, further functional validation is required to confirm this regulatory relationship.

## 4 Discussion

In this study, we found that lower vitamin A levels were associated with more severe sleep problems and autistic symptoms, as well as with altered expression of RARβ and clock genes in children with ASD. In the animal model, we further observed that downregulation of RARβ was associated with changes in brain clock gene expression and autism-like social behaviors. These findings suggest a potential link among vitamin A status, retinoic acid signaling, clock gene expression, and clinical manifestations in ASD, but the causal relationships among these factors remain to be established.

We found that children with lower VA levels exhibited more severe sleep problems, particularly in the dimensions of bedtime resistance. This finding differs from the only previous cross-sectional study examining the VA-sleep relationship in ASD children (9). That study used a CSHQ total score cutoff of 41 (sleep problem group: n=163 vs. normal sleep group: n=18), found no difference in VA levels. The inconsistency may be attributable to differences in grouping strategies and the specific sleep dimensions investigated. The prior study potentially suffered from limitations, including insufficient subscale analysis and statistical bias due to imbalanced group sample sizes. Therefore, our study incorporated a more detailed examination of CSHQ subscale dimensions. Furthermore, a large-scale Japanese study involving more than 3000 participants suggested an association between VA intake and sleep (15), and several population-based studies have reported an association between vitamin A or carotenoid (provitamin A, metabolized to retinol *in vivo*) levels and sleep parameters (16–18), lending support to our findings.

Previous studies have extensively reported the regulatory role of core clock genes in sleep (19, 20). Therefore, we examined the expression levels of BMAL1 and CLOCK in both human and animal models. In human cohorts, we observed a modest time-specific association among retinoic acid signaling, social behavior, and morning clock genes expression. In a mouse model, we similarly found that downregulation of RARβ signaling in the PFC was associated with reduced Clock expression and impaired social ability. The relatively consistent findings from both humans and animals provide preliminary evidence for an interaction among nutrition, clock genes, and neurodevelopment.

Some animal studies have suggested the presence of retinoic acid response elements (RAREs) within the promoter regions of several circadian rhythm molecules, but direct experimental confirmation was lacking (11, 12). Our ChIP-qPCR results indicate RARβ occupancy at a predicted Clock regulatory region and reduced enrichment following RARβ knockdown, which is consistent with a potential regulatory relationship. RARα and RARβ, as the two primary subtypes of nuclear retinoic acid receptors, have similarities in physiological functions. An *in vitro* experiment has demonstrated that RARα can directly interact with the CLOCK protein, potentially modulating circadian rhythms by influencing its heterodimerization with BMAL1 (21). BMAL1, a core clock gene, enhances the stabilization of rhythmic output from the suprachiasmatic nucleus (SCN) when its expression is increased, thereby potentially improving sleep initiation and maintenance (22).

Our study has several limitations. First, the CSHQ questionnaire introduces inherent limitations. It was difficult for us to use objective tests (sleep EEG or somatic sleep recorders) because of time, expense, and the unique nature of children with ASD. This prevented us from accurately assessing the relationship between vitamin A nutritional status and sleep. Second, peripheral blood clock gene mRNA levels may not fully reflect changes in circadian rhythm molecules within the central nervous system. Third, blood samples from patients and brain tissue from mice were both collected at a single morning time point, which precluded assessment of circadian rhythmicity. Finally, technical limitations prevented us from monitoring sleep-wake cycles or sleep phenotypes in animal models through behavioral experiments, making it impossible to verify the causal relationship between retinoic acid signaling and circadian rhythms or sleep in animals. Future studies should further explore these associations through longitudinal follow-up, multi-time point sampling, and objective sleep assessments in both humans and animal models.

## 5 Conclusion

This study is the first to explore the associations among retinoic acid signaling, sleep, and ASD, and to examine the expression of RARβ and specific clock genes in morning peripheral blood samples. The results showed that lower vitamin A levels were associated with more severe sleep problems, greater symptom burden, and altered clock gene expression. RARβ knockdown led to reduced Clock expression and impaired social ability. Although these findings cannot establish causality, they provide a preliminary framework for future longitudinal studies and further mechanistic exploration.

## Data Availability

The original contributions presented in the study are included in the article/**Supplementary Material**. Further inquiries can be directed to the corresponding author.
