# Supplementary material for: Vitamin A status is associated with sleep, clock genes, and symptoms in children with autism spectrum disorder
Source: Front Psychiatry. 2026 Apr 7;17:1805599. doi: 10.3389/fpsyt.2026.1805599 (PMC13095683; doi:10.3389/fpsyt.2026.1805599)
Supplement: Supplementary file 1 [file Table1.docx]

Supplementary Material

# Table S1. Primer sequence

| **Name** |  | **Sequence** |
| --- | --- | --- |
| BMAL1 | Fwd | 5'-TTACTGTGCTAAGGATGGCTGTTC-3’ |
|  | Rev | 5'-AGTTGGTTTGTAGTTTGCTTCTGTG-3’ |
| CLOCK | Fwd | 5'-GTGACCAAATTAGTGACTGCTCCTG-3’ |
|  | Rev | 5'-GCTGCTGCTGCTGCGTTAC-3’ |
| RARβ | Fwd | 5'-CCGCCTGCCTGGACATCC-3’ |
|  | Rev | 5'-GTGAACACAAGGTCAGTCAGAGG-3’ |
| β-actin | Fwd | 5'-GTGAAGGTGACAGCAGTCGGTT-3’ |
|  | Rev | 5'-GAGAAGTGGGGTGGCTTTTAGGA-3’ |

# Table S2. Primer sequence in mice

| Name |  | Sequence(5'-3’) in SH-SY5Y | Sequence(5'-3’) in C57 |
| --- | --- | --- | --- |
| RARβ | Fwd | CCGCCTGCCTGGACATCC | CAATGCTGGCTTCGGTCCTCTG |
|  | Rev | GTGAACACAAGGTCAGTCAGAGG | CCTCAAGGTCCTGGCGGTCTC |
| BMAL1 | Fwd | TTACTGTGCTAAGGATGGCTGTTC | AGCCCGCTGAACATCACAAGTAC |
|  | Rev | AGTTGGTTTGTAGTTTGCTTCTGTG | TGAGCCTGCCCTGGTAATAGTCC |
| CLOCK | Fwd | GTGACCAAATTAGTGACTGCTCCTG | GCCAGCCACCACAGCAGTTC |
|  | Rev | GCTGCTGCTGCTGCGTTAC | GTGGAAAGGCAGCAGAGAGGATG |
| RORA | Fwd | TCAGGAGAAGTCAGCAAAGCAATG | CTTGTACGCCGAGGTGCAGAAG |
|  | Rev | GACAGTGTTGGCAGCGGTTTC | GCAGTTCCGTCAGCCCATTGG |
| REVERBα | Fwd | TCCTCATCTTCCTCGTCGTCATC | CTTCCTCCTACCCGCCTACCTG |
|  | Rev | ACACAGTAACACCATGCCATTCAG | TGTTGCCTTGCCGTAGACTGTTG |
| PER1 | Fwd | ACCAAGAAAGATCCGCCGTCAG | ACTGTGGCAGCAGCGTTCAAG |
|  | Rev | ACACCACACTCTCCGCCTTATTG | CCTCGCCTCCACCCTCTTCC |
| PER2 | Fwd | GAAGCAGGTGAAAGCCAATGAAGAG | GCTGCGGATGCTCGTGGAATC |
|  | Rev | TCTCCATCTCCTCCACGGTGTAG | GGTTGTGCTCTGCCTCTGTCATC |
| CRY1 | Fwd | GTTGGATGTGGCTGTCTTGTAGTTC | TTCTCGCCTCGGTCCCTTCTAAC |
|  | Rev | AACGCCTGATATAGTCTCCATTGGG | AGCCCATGAGCCCTCCATTCC |
| CRY2 | Fwd | TGGATAAGCACTTGGAACGGAAGG | TGGACAAGCACTTGGAACGGAAG |
|  | Rev | TAGTAGAAGAGGCGGCAGGAGAG | GTAGAAGAGGCGGCAGGAGAGG |
| β-actin | Fwd | GTGAAGGTGACAGCAGTCGGTT | CACGGCAAATTCAACGGCACAG |
|  | Rev | GAGAAGTGGGGTGGCTTTTAGGA | TCGCTCCTGGAAGATGGTGATGG |

# Table S3. Predicted binding site of mouse RARβ on the promoter of Clock by JASPAR

| Name | Sequence | Strand | Relative score |
| --- | --- | --- | --- |
| RARβ | AGGTGACTAAGAGGACA | + | 0.805 |
| RARβ | AAGGGGGCAAAGGTCA | + | 0.797 |

# Table S4. Primer sequence in ChIP

| Name |  | Sequence (5'-3’) |
| --- | --- | --- |
| Clock-1 | Fwd | TCGTAAACTGAACCGTGGGA |
|  | Rev | TGCCCCCTTTAATGGTTTCAT |
| Clock-2 | Fwd | GTGACAGAGGACTGAGCAGAA |
|  | Rev | CTTTGCCCCCTTTAATGGTTTCAT |

**
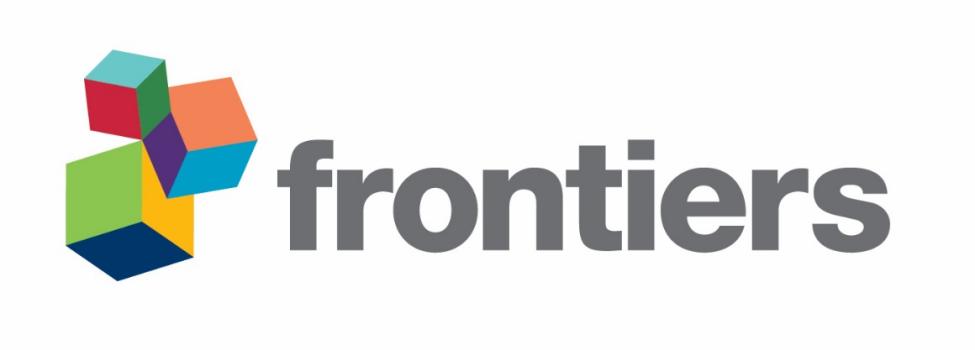
**
